# Draft Genome Sequences of Type VI Secretion System-Encoding Vibrio fischeri Strains FQ-A001 and ES401

**DOI:** 10.1128/MRA.00385-19

**Published:** 2019-05-16

**Authors:** Katherine M. Bultman, Andrew G. Cecere, Tim Miyashiro, Alecia N. Septer, Mark J. Mandel

**Affiliations:** a Department of Medical Microbiology and Immunology, University of Wisconsin–Madison, Madison, Wisconsin, USA; b Department of Biochemistry and Molecular Biology, Pennsylvania State University, University Park, Pennsylvania, USA; c Department of Marine Sciences, University of North Carolina at Chapel Hill, Chapel Hill, North Carolina, USA; University of Maryland School of Medicine

## Abstract

The type VI secretion system (T6SS) facilitates lethal competition between bacteria through direct contact. Comparative genomics has facilitated the study of these systems in Vibrio fischeri, which colonizes the squid host Euprymna scolopes. Here, we report the draft genome sequences of two lethal V. fischeri strains that encode the T6SS, FQ-A001 and ES401.

## Abstract (precis)

The type VI secretion system (T6SS) facilitates lethal competition between bacteria through direct contact. Comparative genomics has facilitated the study of these systems in Vibrio fischeri, which colonizes the squid host Euprymna scolopes.

## ANNOUNCEMENT

The bacterially encoded type VI secretion system (T6SS) is a membrane-embedded syringe-like structure that delivers effectors to bacterial and, in some cases, eukaryotic cells (1). A role for the T6SS has been demonstrated in mediating strain separation of incompatible Vibrio fischeri strains during colonization of *Euprymna scolopes* juvenile squid (2). V. fischeri FQ-A001 (3) and ES401 (4) are strains that were isolated decades apart, yet both exhibit the lethal phenotype, defined as the ability to kill V. fischeri ES114 in direct competition (2). Experimental analysis of FQ-A001 has demonstrated that a specific T6SS locus (T6SS2) is responsible for the observed lethal phenotype (2). Draft genomes for FQ-A001 and ES401, both of which have an intact T6SS2 locus, were obtained as follows.

Strain FQ-A001 was isolated from an E. scolopes adult female (21-mm mantle length) from Kaneohe Bay, Oahu, HI, in 2015, and was cultivated in the laboratory on LB salt (LBS) medium (3). For Illumina MiSeq sequencing (2 × 250 bp; Penn State Genomics Core Facility), genomic DNA was isolated using the MasterPure DNA purification kit (Epicentre, Madison, WI), and the library was constructed using the TruSeq DNA PCR-free kit (Illumina, San Diego, CA). For PacBio RS II single-molecule real-time (SMRT) sequencing (UNC Chapel Hill High-Throughput Sequencing Facility), genomic DNA was isolated by phenol-chloroform extraction, and the sequencing library was prepared using the SMRTbell library prep kit (10-kb size selection). Approximately 9.4 × 10^8^ bp (MiSeq) and 1.1 × 10^9^ bp (RS II) of sequence data were obtained, yielding approximately 470-fold coverage of the FQ-A001 genome. *De novo* assembly of the FQ-A001 genome was conducted using SPAdes version 3.13.0 (with the parameters “-careful -k 127”) (5), yielding four contigs of >1,000 bp. Contigs were reordered using Mauve Contig Mover, with the ES114 genome as a reference (6–8). For exploratory analysis, the genome was annotated with Prokka 1.13.3 (9), and the NCBI Prokaryotic Genome Annotation Pipeline (PGAP; accessed 4 March 2019) was used to annotate the contigs for deposition to GenBank (10).

Strain ES401 was isolated from an *E. scolopes* juvenile (3.5-mm mantle length) from Maunalua Bay, Oahu, HI, in 1990 and can be cultivated in the laboratory on LBS medium (4). For Illumina MiSeq sequencing (2 × 300 bp; University of Wisconsin Biotechnology Center), genomic DNA was isolated using the Gram-negative bacterial protocol in the DNeasy blood and tissue kit (Qiagen USA, Germantown, MD), and the library was constructed using the TruSeq Nano kit (Illumina, San Diego, CA). PacBio sequencing was conducted as described above for FQ-A001. Approximately 2.7 × 10^8^ bp (MiSeq) and 1.8 × 10^9^ bp (RS II) of sequence data were obtained, yielding approximately 450-fold coverage of the ES401 genome. Assembly, annotation, and GenBank deposition of the resulting 6 contigs of >1,000 bp were conducted as described above for FQ-A001 but with the SPAdes parameters “-careful -k 125” and with PGAP (accessed 2 April 2019).

For quality control, the original Illumina and PacBio base calling resulted in 0 sequences flagged as poor quality by FastQC (http://www.bioinformatics.babraham.ac.uk/projects/fastqc). Upon submission to the PGAP, an additional fifth contig in the FQ-A001 sequence contained only the PhiX phage sequence and was excluded from the assembly and further analysis. PGAP additionally identified an Illumina adapter sequence at one location in the ES401 genome; we conducted Sanger sequencing of this region, which clarified the correct sequence. The correct sequence was resubmitted to PGAP, and the surrounding genomic context was not affected.

Analysis using progressiveMauve (snapshot 2015_02_25, macOS) revealed genomes that are colinear with MJ11 and the presence of the lethal strain-specific T6SS on chromosome II in both FQ-A001 and ES401 (Table 1) (2, 11, 12). Default parameters for software were used except where additional parameters are noted above.

**TABLE 1:** Vibrio fischeri genomes described in this report

Strain	Yr isolated	Genome size (bp)	No. of contigs	*N*_50_	Total no. of genes	G+C content (%)	GenBank accession no.	Genes corresponding to MJ11 T6SS2
FQ-A001	2015	4,286,900	4	2,417,280	4,015	38.3	SJSX00000000	VFFQA001_15465 to VFFQA001_15635
ES401	1990	4,282,653	6	1,351,222	4,011	38.3	SRJG00000000	VFES401_15680 to VFES401_15865

## 

### Data availability.

The data for this paper are available at the NCBI as FQ-A001 genome accession number SJSX00000000 (the version reported here is the first version, SJSX01000000), FQ-A001 read accession numbers SRR8647323 and SRR8647324, ES401 genome accession number SRJG00000000 (first version, SRJG01000000), and ES401 read accession numbers SRR8708068 and SRR8708069.
